# Supplementary material for: Transmembrane protein 97 is a potential synaptic amyloid beta receptor in human Alzheimer’s disease
Source: Acta Neuropathol. 2024 Feb 6;147(1):32. doi: 10.1007/s00401-023-02679-6 (PMC10847197; doi:10.1007/s00401-023-02679-6)
Supplement: Supplementary file 1 — Supplementary file1 (PDF 3289 KB) [file 401_2023_2679_MOESM1_ESM.pdf]

Supplementary materials

TMEM97 increases in synapses and is a potential synaptic Aβ binding partner in human Alzheimer's disease

Martí Colom-Cadena *et al.*

Table S1. Mice included in array tomography-FRET experiments.

Table S2. Antibodies.

Fig. S1. Image analysis pipeline.

Fig. S2. Characterization of soluble Aβ in human brain samples.

Fig. S3. Effect of sigma-2 receptor antagonist on PSD95, Aβ and tau in the non-transgenic control mice and APP/PS1+Tau mice.

Fig. S4. Plaque and astrocyte burdens in transgenic mice treated with CT1812 or vehicle.

Fig. S5. Functional imaging of iPSC neurons treated with CT1812 or vehicle.

Table S1. Mice included in array tomography-FRET experiments.

Case	Animal ID	Treatment	Age at death	Sex	Brain CT1812 (ng/mL)	Blood CT1812 (ng/mL)	Estimated receptor occupancy (%)
1	G353	vehicle	10,2	f	0	NA	0
2	G394	vehicle	10,1	f	0	0	0
3	G395	vehicle	10,1	f	0	0	0
4	G439	vehicle	9,9	f	0	0	0
5	G443	vehicle	9,9	m	0	0	0
6	G454	vehicle	9,9	m	0	0	0
7	G455	vehicle	9,9	m	0	0	0
8	G458	vehicle	9,9	m	0	0	0
9	G362	CT1812	10,1	f	9,7	0,9	72,5
10	G365	CT1812	10,1	f	9,0	0,7	71,0
11	G372	CT1812	9,9	m	12,3	0,9	77,0
12	G413	CT1812	10,1	f	18,8	1,1	83,7
13	G421	CT1812	10,0	m	22,0	0,1	85,7
14	G429	CT1812	9,9	m	47,5	7,1	92,8
15	G435	CT1812	9,9	m	15,4	NA	80,7
16	G444	CT1812	9,9	m	30,9	1,9	89,4
17	G519	CT1812	9,9	f	7,9	0,5	68,4
18	G520	CT1812	9,9	f	4,1	0,2	52,9

Abbreviations: f, female; m, male; NA, not available.

Table S2. Antibodies.

Primary Antibodies	Host specie	Reactivity specie	Supplier	Catalogue #	Dilution	Study
PSD95	Guinea Pig	Human	Synaptic Systems	124-014	1:50	AT-FRET main study
Aβ (6E10)	Mouse	Human	Biolegend	39320	1:200	AT-FRET main study
TMEM97	Rabbit	Human	Novus Biologicals	NBP1-30436	1:100	AT-FRET main study
NAB61	Mouse	Human	collaborator	n/a	1:50	AT- FRET other interactors
PrPc (EP1802Y)	Rabbit	Human	Abcam	ab52604	1:50	AT- FRET other interactors
Synaptophysin (Sy38)	Mouse	Human	Abcam	Ab8049	1:50	AT-FRET other interactors
Synaptophysn	Goat	Human	AF5555	R&D Systems	1:50	AT-FRET other interactors
PSD95	Rabbit	Human	Cell Signaling	D27E11	1:50	AT-FRET other interactors
PGRMC1	Goat	Human	Abcam	Ab48012	1:50	AT-FRET other interactors

Secondary Antibodies	Host specie	Reactivity specie	Supplier	Catalogue #	Dilution	Study
Alexa Fluor 488®	Donkey	Guinea Pig	Jackson Immuno	706-545-148	1:50	AT-FRET main study
Cy™3	Donkey	Mouse	Jackson Immuno	715-165-150	1:50	AT-FRET main study
Cy™5	Donkey	Rabbit	Jackson Immuno	711-175-152	1:50	AT-FRET main study
Alexa Fluor 488®	Donkey	Mouse	Abcam	ab150105	1:50	AT-FRET other interactors
Alexa Fluor 405®	Donkey	Guinea Pig	Jackson Immuno	706-475-148	1:50	AT-FRET other interactors
Alexa Fluor 568®	Donkey	Rabbit	Abcam	ab175470	1:50	AT-FRET other interactors
Alexa Fluor 647®	Donkey	Goat	Abcam	ab150135	1:50	AT-FRET other interactors
Cy™3	Goat	Mouse	Jackson Immuno	115-165-146	1:50	AT-FRET positive control
Cy™5	Donkey	Goat	Jackson Immuno	705-175-147	1:50	AT-FRET positive control

Fig. S1. Image analysis pipeline.

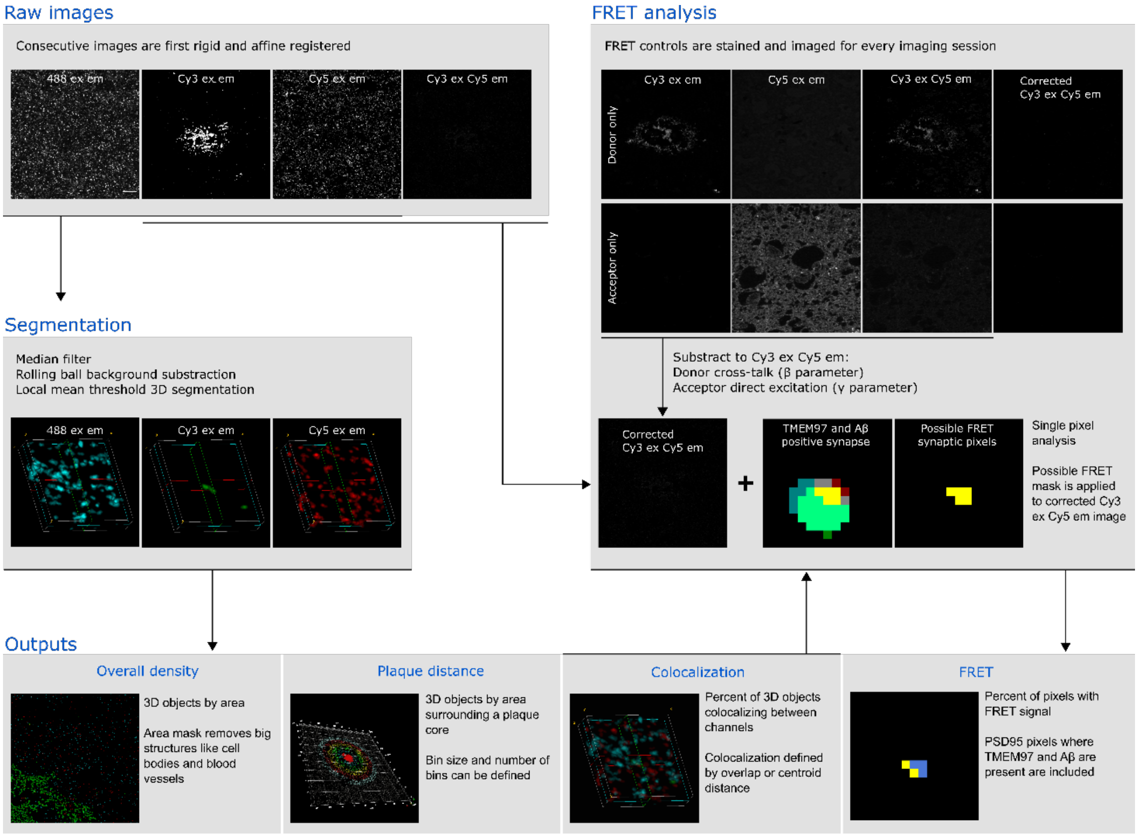

The diagram describes the basic image analysis steps performed in the current study. All the processes were combined into an in-house algorithm available at: https://github.com/Spires-Jones-Lab

Fig. S2. Characterization of soluble Aβ in human brain samples.

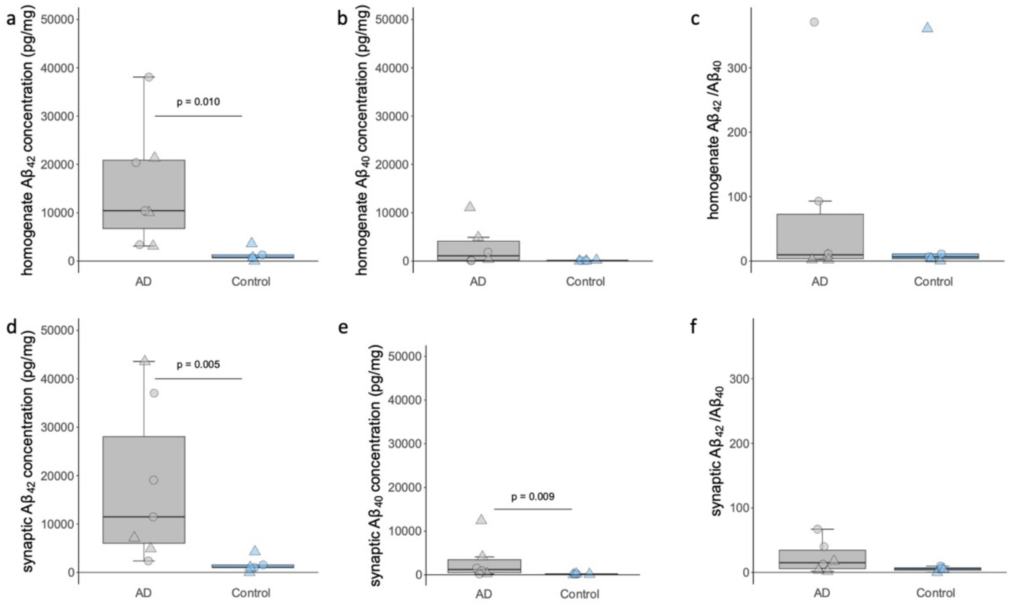

Both Aβ42 (**a, d**) and Aβ40 (**b, e**) are detected by ELISA in soluble homogenates and synaptoneurosome fractions from AD (n=6) and control brain (n=6). Levels of Aβ42 are higher in AD brain in both fractions, while levels of Aβ40 are only significantly elevated in synaptic fractions. The ratio of Aβ42/Aβ40 (**c, f**) is not changed in either fraction. P values are from Wilcoxon tests. When the measured value was below the detection limit of the ELISA, a value of 10pg/mg (the lower detection limit) was used.

Fig. S3. Effect of sigma-2 receptor antagonist on PSD95, Aβ and tau in the non-transgenic control mice and APP/PS1+Tau mice.

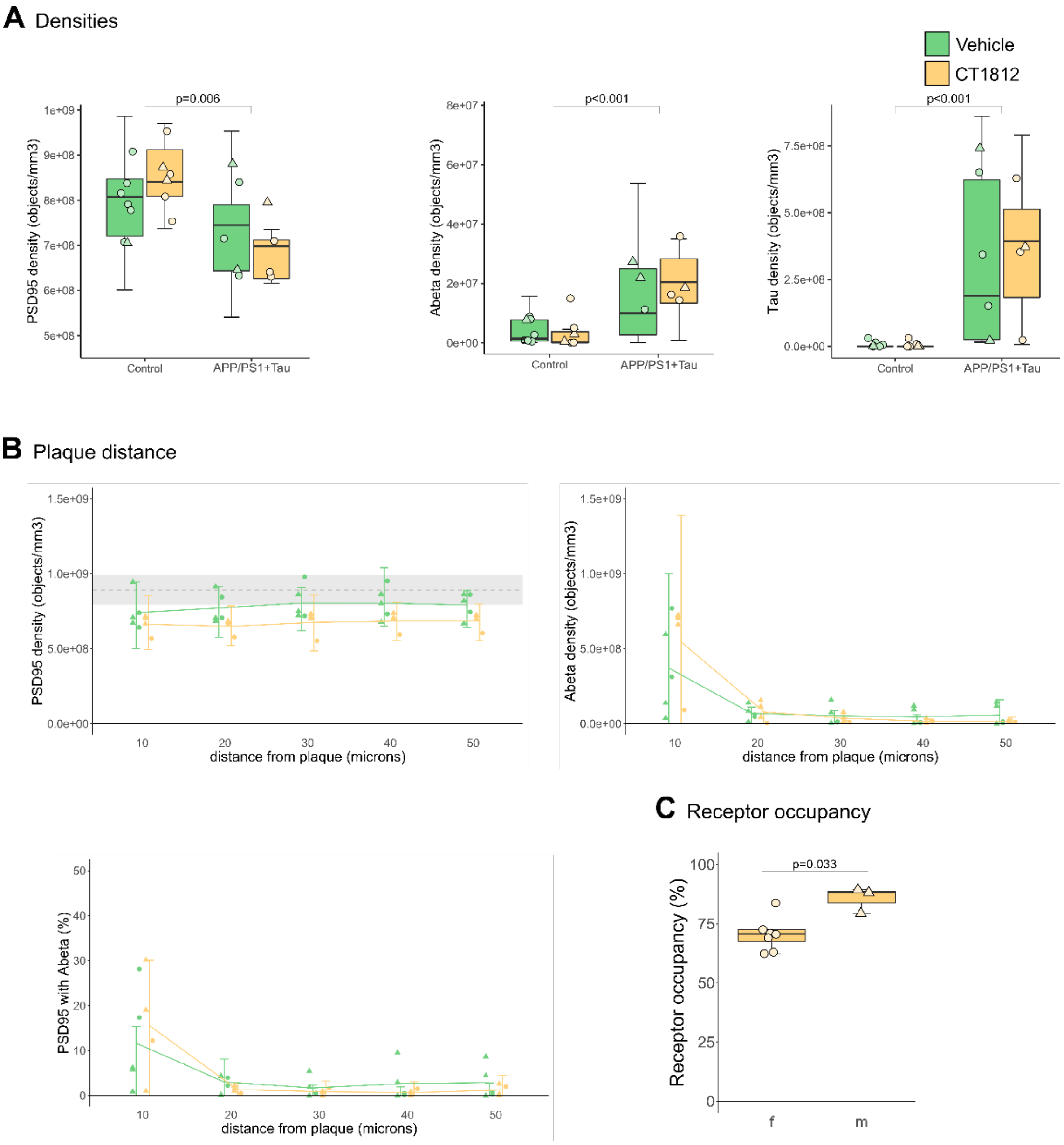

In **A** are quantified the densities of object found for either PSD95, Aβ or tau in the non-transgenic control mice and the APP/PS1+Tau mice model. In **B**, plaque distance density of either PSD95, Aβ, or PSD95 that contain Aβ in the APP/PS1+Tau are plotted. Grey dotted line show the mean of control mice and the SD is shown in grey. Panel **C** shows the estimated receptor occupancy by the drug in the treated mice, independently of the genotype. Boxplots show quartiles and medians calculated from each image stack. Data points refer to case means (females, triangles; males, circles, green = vehicle, orange = CT1812). Analysis with linear mixed effects models including treatment group and sex interaction. Abbreviations: f, female; m, male.

Fig. S4. Plaque and astrocyte burdens in transgenic mice treated with CT1812 or vehicle.

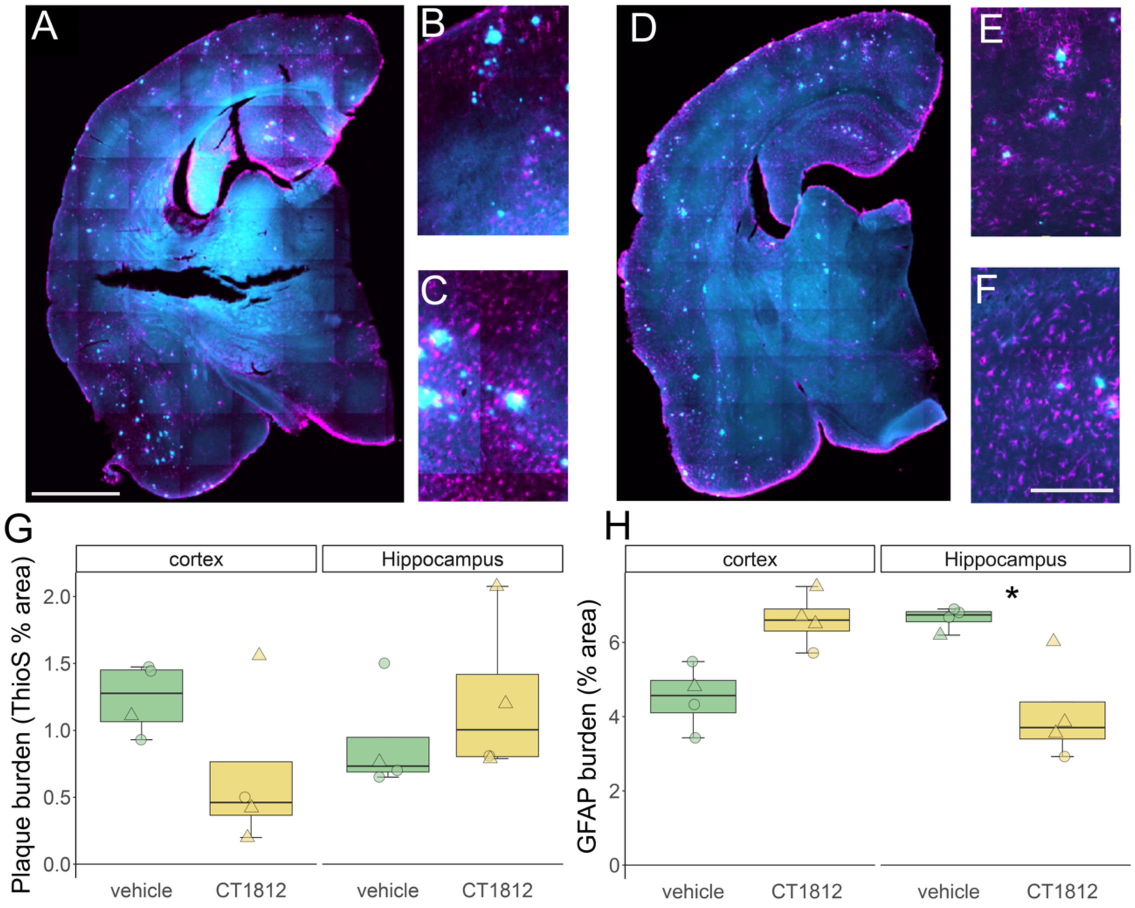

Thioflavin S plaque burden and GFAP astrocyte burden were examined on 50 micrometer floating sections (3 per mouse) in animals treated with vehicle (A-C) or CT1812 (only those with >80% estimated receptor occupancy included in the study, representative images D-F). Tilescans of the entire section were taken (A, D) and analysis carried out in cortex (B, E) and hippocampus (C,F). While there were no significant differences in plaque burdens (G), ANOVA on linear mixed effects models of astrocyte burdens shows a significant interaction between treatment and brain region ($F[1,16]=9.46$, $p=0.007$) and a significant post-hoc difference decrease in astrocyte burden in hippocampus with CT1812 treatment ($t=2.43$, $p=0.03$). Scale bars represent 1mm in A and D and 250 μm in B, C, E, and F

Fig. S5. Functional imaging of iPSC neurons treated with CT1812 or vehicle.

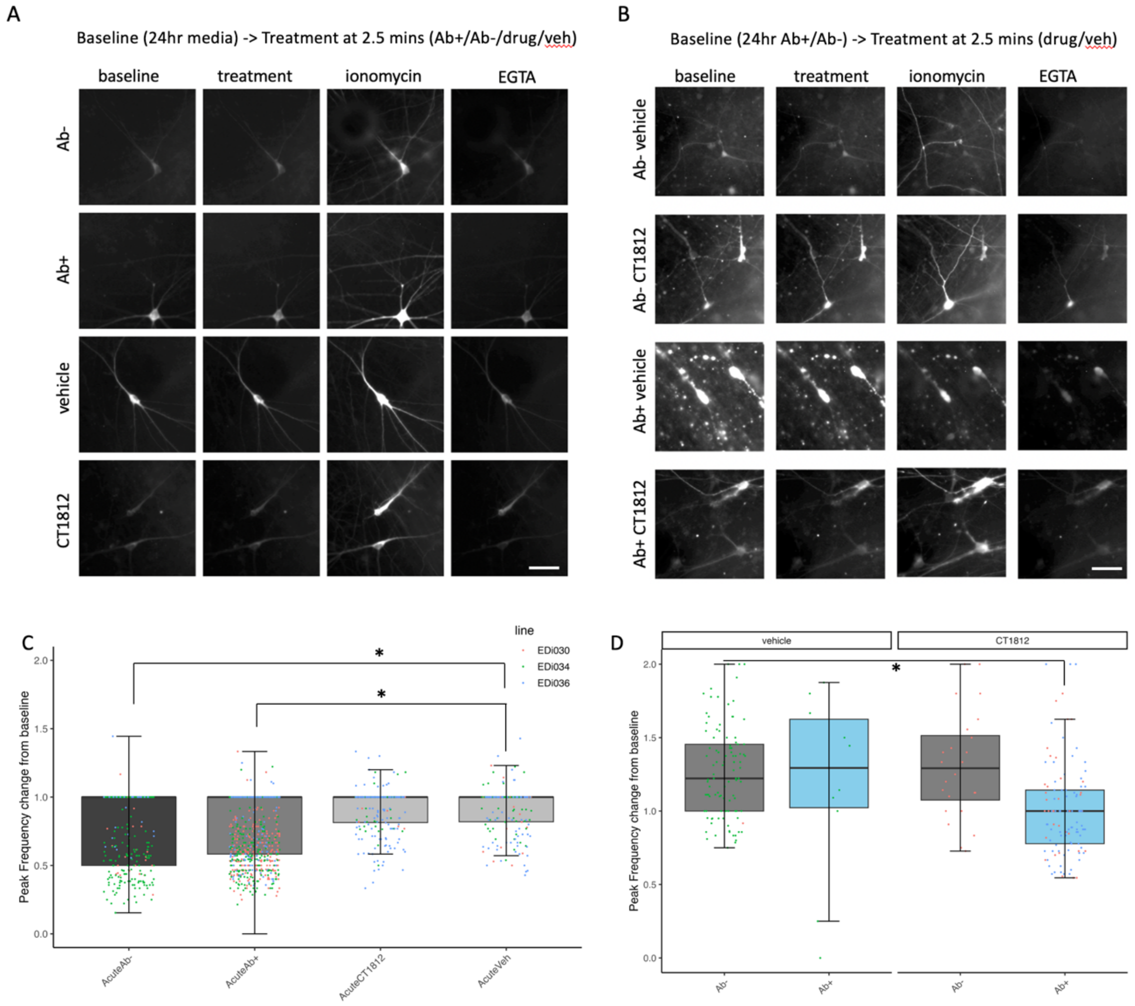

GCaMP6 virus was incubated for 1 week with iPSC derived neurons at approximately day 173-180 post differentiation. Cells were either treated acutely (A, C) with homogenate (Aβ+ or Aβ+), 10 μM CT1812, or vehicle followed by positive and negative controls ionomycin and EGTA to activate or inhibit activity; or they were pre-treated for 24 hours (B, D) with homogenate (Aβ+ or Aβ+) followed by baseline imaging then treatment with 10 μM CT1812, or vehicle followed by positive and negative controls. Images of GCaMP6 fluorescence (A, B) show that neurons were active in all conditions (Scale bars represent 50 μm). Quantification of the peak frequency of GCaMP normalized to the baseline condition for each group shows that acute treatment with homogenate or drug does not change between Aβ- or Aβ+ homogenate or CT1812 or vehicle (C). However, both human brain homogenate treated groups are lower than the control (vehicle treatment; Tukey-corrected post hoc test after linear mixed effects model, $p<0.0001$) indicating that exposure to human brain homogenate affects cell function. Pre-incubation with homogenate for 24 hours before CT1812 or vehicle treatment (D) shows that there are significant effects of drug treatment (ANOVA after linear mixed effects model $F[1,257]=9.15$, $p=0.003$) and a significant interaction between Aβ status of the homogenate and drug treatment ($F[1,257]=9.06$, $p=0.003$). There is a significant decrease in peak frequency between Aβ– incubation followed by vehicle treatment and Aβ+ followed by CT1812 treatment (*). In graphs, each line from a single donor is represented by a different colour. In C and D, each point represents peak frequency of individual objects measured.
